# Treatment at Health Resorts

**Published:** 1906-07-07

**Authors:** 


### Treatment at Health Resorts.

In his interesting address recently delivered at Cheltenham, Dr. Goodhart discussed with the saving common sense that marks all his utterances some of the problems associated with the therapeutic influences and values of health-resorts. The subject has an importance which, it is to be feared, is by no means fully appreciated by the medical profession in this country. To many the whole question resolves itself into an affair of custom and fashion associated for the time being with a more or less chastened obedience to the elementary rules of hygiene. The suggested appreciation of minute climatic differences and specific values they regard with suspicion and disdain, and occasional utterances of professed experts doubtless to some extent justify this attitude. Yet, beyond question, there is a large body of practical testimony to the health-promoting influence of various localities and of natural mineral waters. It cannot be wise to dismiss all this as an expression of the removal of imagined ills, or to assume that there is nothing more in it than the result of a wisely-spent holiday. Rather it is in the interests of sound and successful medical practice that the conclusions suggested by so emphatic a popular verdict should be critically tested and examined, and it is to be hoped that Dr. Goodhart's exhortation to this study will meet with a fruitful result. Those who practise medicine at health-resorts may be expected not unnaturally to view with a favourable eye the virtues of climatic treatment generally, and in particular the value of the district with which they happen to be associated. To some extent, therefore, their testimony is looked upon as prejudiced, and is to a corresponding degree weakened. And so long as it consists of more or less vague statements with lists of therapeutic successes no additional strength can be gained. What is wanted is some more tangible evidence, the evidence of facts and figures than can be weighed and measured. There are many difficulties to overcome, but the possibilities to the patient worker in this direction are well illustrated by the results accomplished by Dr. George Oliver, which include substantial contributions to our knowledge of the physiology and pathology of the cardio-vascular mechanism. It cannot be doubted that were more evidences of what may be termed exact medicine to issue from those practising in health stations there would be in corresponding degree a growing appreciation of the value of climatic and allied forms of treatment among the general body of the profession.

Another influence needed to establish confidence in the specific value of climates and spas in the treatment of disease is an increased recognition of the reality of disorders that give no evidence of their existence in the shape of physical signs. The young clinician fresh from the triumphs of the schools is apt to treat with incredulity the suggestion of functional disease and to regard as non-existent what cannot be demonstrated by physical and chemical methods. Equally the busy practitioner has to deal with so many minor maladies which either disappear in an unexplained fashion or remain as the burden of a lifetime, that he may readily fail to realise that some disturbance of the subtle processes of tissue chemistry may be at the bottom of a great deal of chronic illness, and may conceivably be readily rectified by a course of appropriate waters. These and many others may read with advantage Dr. Goodhart's profession of faith that in persons after 50 years of age or so the bodily organs do not work so automatically towards the right or so naturally make for health as is the case in earlier years. With this inevitable tendency the tissues produce products of imperfect combustion and consequently the blood and other fluids become charged with impurities. Whether all this is or is not "gout" is a matter largely for academic debate. The important fact is that these disturbances exist, that they are responsible for a great deal of what the sufferers regard as disease, however the profession may view it, and that it is not unreasonable to suppose that mineral waters wisely selected may restore the balance in favour of health. But before this conclusion is reached there is the essential preliminary conviction that medical practice deals with a quantity of ill-defined disorder which it is difficult or impossible to classify and which has a real basis in the shape of disturbed function. When this is recognised it is at least possible to anticipate that the general and specific influences of a wisely selected health station may be both a scientific and successful therapeutic measure. And from this position it is not difficult to advance to the claim that such a prescription should not be a mere "change of air" but should be set on definite lines and guided by some definite principle.

Those who read Dr. Goodhart's excellent address will find much more than we can possibly allude to here. But there are two practical points which we cannot omit. The one is that arrangements should be made at our home stations for visitors to obtain some simpler forms of diet than that comprised in the usual hotel menu. The other that physicians at health-resorts should try so far as possible to get the results of the mineral waters they prescribe without the confusing influences of other drugs. If these and other counsels from the same authority are adopted the scope of climatic and spa treatment in this country may be much extended with great advantage to all concerned.

